# AI-based automation of enrollment criteria and endpoint assessment in clinical trials in liver diseases

**DOI:** 10.1038/s41591-024-03172-7

**Published:** 2024-08-07

**Authors:** Janani S. Iyer, Dinkar Juyal, Quang Le, Zahil Shanis, Harsha Pokkalla, Maryam Pouryahya, Aryan Pedawi, S. Adam Stanford-Moore, Charles Biddle-Snead, Oscar Carrasco-Zevallos, Mary Lin, Robert Egger, Sara Hoffman, Hunter Elliott, Kenneth Leidal, Robert P. Myers, Chuhan Chung, Andrew N. Billin, Timothy R. Watkins, Scott D. Patterson, Murray Resnick, Katy Wack, Jon Glickman, Alastair D. Burt, Rohit Loomba, Arun J. Sanyal, Ben Glass, Michael C. Montalto, Amaro Taylor-Weiner, Ilan Wapinski, Andrew H. Beck

**Affiliations:** 1 PathAI, Boston, MA USA; 2 Gilead Sciences, Inc., Foster City, CA USA; 3 NIHRB Medical Research Center, Newcastle University, Newcastle, UK; 4 NAFLD Research Center, Division of Gastroenterology and Hepatology, University of California at San Diego, San Diego, CA USA; 5 Stravitz-Sanyal Institute for Liver Disease and Metabolic Health, VCU School of Medicine, Richmond, VA USA; 6 Present Address: Absci, Vancouver, WA USA; 7 Present Address: AstraZeneca, Gaithersburg, MD USA; 8 Present Address: Atomwise, San Francisco, CA USA; 9 Present Address: Johnson & Johnson, New Brunswick, NJ USA; 10 Present Address: Supernus Pharmaceuticals, Rockville, MD USA; 11 Present Address: Harvard Medical School, Boston, MA USA; 12 Present Address: BigHat Biosciences, San Mateo, CA USA; 13 Present Address: Genesis Therapeutics, Burlingame, CA USA; 14 Present Address: OrsoBio, Inc., Palo Alto, CA USA; 15 Present Address: Inipharm, San Diego, CA USA; 16 Present Address: Rhode Island Hospital and The Miriam Hospital, Providence, RI USA; 17 Present Address: Massachusetts General Hospital, Boston, MA USA; 18 Present Address: Amgen, Thousand Oaks, CA USA; 19 Present Address: Sanofi Pharmaceuticals, Cambridge, MA USA

**Keywords:** Non-alcoholic steatohepatitis, Machine learning

## Abstract

Clinical trials in metabolic dysfunction-associated steatohepatitis (MASH, formerly known as nonalcoholic steatohepatitis) require histologic scoring for assessment of inclusion criteria and endpoints. However, variability in interpretation has impacted clinical trial outcomes. We developed an artificial intelligence-based measurement (AIM) tool for scoring MASH histology (AIM-MASH). AIM-MASH predictions for MASH Clinical Research Network necroinflammation grades and fibrosis stages were reproducible (*κ* = 1) and aligned with expert pathologist consensus scores (*κ* = 0.62–0.74). The AIM-MASH versus consensus agreements were comparable to average pathologists for MASH Clinical Research Network scores (82% versus 81%) and fibrosis (97% versus 96%). Continuous scores produced by AIM-MASH for key histological features of MASH correlated with mean pathologist scores and noninvasive biomarkers and strongly predicted progression-free survival in patients with stage 3 (*P* < 0.0001) and stage 4 (*P* = 0.03) fibrosis. In a retrospective analysis of the ATLAS trial (NCT03449446), responders receiving study treatment showed a greater continuous change in fibrosis compared with placebo (*P* = 0.02). Overall, these results suggest that AIM-MASH may assist pathologists in histologic review of MASH clinical trials, reducing inter-rater variability on trial outcomes and offering a more sensitive and reproducible measure of patient responses.

## Abstract (web summary)

Using data from MASH clinical trials, an AI model shows comparable performance with respect to individual pathologists in assessing histologic scoring for enrollment criteria and endpoint evaluation

## Main

Metabolic dysfunction-associated steatohepatitis (MASH), formerly known as nonalcoholic steatohepatitis, is the progressive form of metabolic dysfunction-associated steatotic liver disease (MASLD), formerly nonalcoholic fatty liver disease. MASH is a frequent cause of cirrhosis and hepatocellular carcinoma and is the most common indication for liver transplantation in women and older adults in the United States^1^. MASH, as well as cirrhosis caused by this disease, has been increasing in incidence^1^, leading to medical and economic burden^2^. Notably, resmetirom was recently the first therapeutic granted regulatory approval for the treatment of MASH^3^.

Histologic surrogate endpoints are currently accepted in MASH clinical trials. Histologic criteria reflecting disease activity or severity are used as the basis for trial enrollment, risk stratification and endpoint assessment. However, limited sensitivity of scoring systems and variability in manual assessment of histology-based endpoints can contribute to incomplete measurement of treatment response^4,5^, clinical trial failure^6^, difficulty in identifying an appropriate study population, and unintended inclusion or exclusion of study participants^6^. Such errors could affect observed treatment responses and trial safety.

The US Food and Drug Administration (FDA) and the European Medicines Agency (EMA) have issued guidance on the use of histopathologic assessment of liver biopsies as clinical trial inclusion criteria and endpoints to measure trial outcomes to support accelerated approval for MASH therapeutics^7^. Similar to most histologic scoring systems proposed to date, the MASH Clinical Research Network (CRN), used by the majority of studies and accepted by both the FDA and EMA, recommends measurement of four key features: macrovesicular steatosis, lobular inflammation, hepatocellular ballooning and fibrosis^8–10^. Despite ongoing efforts by liver pathologists with expertise in MASH histology to harmonize scoring guidelines^11^ in clinical trials and real-world settings^9,11–13^, a recent study reported that a substantial portion of a MASH clinical trial cohort did not meet enrollment criteria upon re-evaluation by a second hepatopathologist^6^. In addition, high variability has been reported between pathologists in the identification of ballooned hepatocytes^12^. This lack of reliability can reduce the power of MASH trials to detect a significant drug effect, as trials are not typically powered to adequately account for such scoring variability.

Advances in artificial intelligence (AI) have led to the development of algorithms that can enable accurate, quantitative and reproducible assessment of digitized pathology whole-slide images (WSIs)^5,14^. However, these algorithms are not yet employed in clinical settings and have not received regulatory approval for clinical trial use. Here, we report a robust approach to evaluate MASH disease severity and improve clinical trial reliability using an AI-powered digital pathology tool—referred to as ‘AIM-MASH’—to quantify relevant histological tissue features.

## Results

### Overview of model-based evaluation of MASH histology

**Fig. 1:**
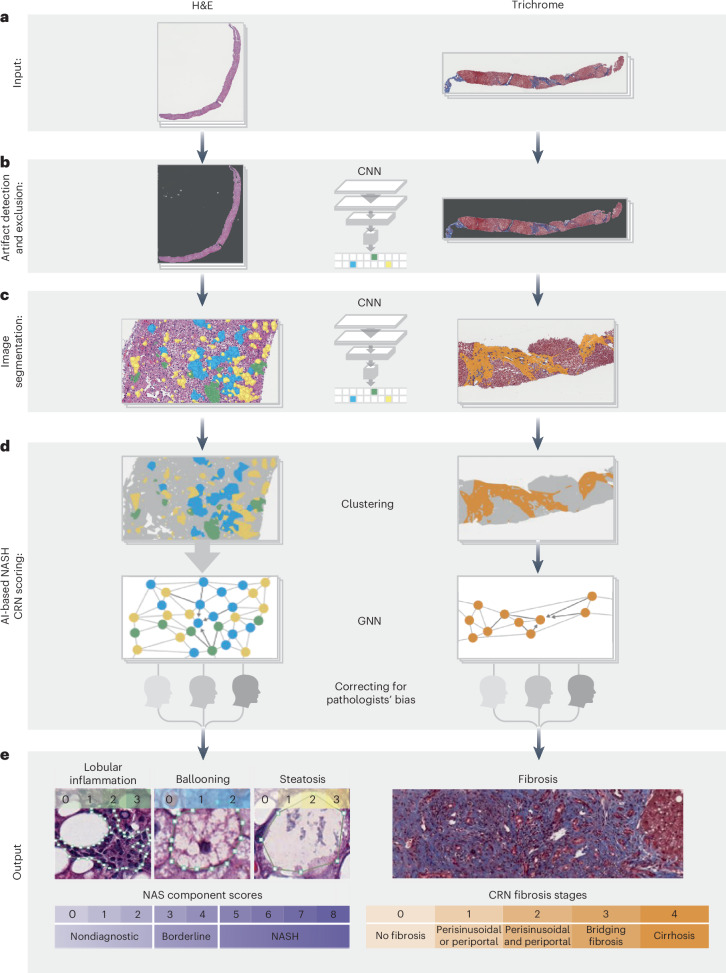
Pipeline for model deployment. **a**, Input: separate CNN-based models trained with digitized H&E- and MT-stained images annotated by expert pathologists are deployed on H&E- or MT-stained WSIs, respectively, to identify histological features. **b**, Artifact detection and exclusion: an artifact model, also based on CNNs, detects image and tissue artifacts for both H&E and MT WSIs and excludes them before downstream analysis. **c**, Image segmentation: H&E and MT CNNs segment and generate pixel-level predictions of relevant histologic features. **d**, AI-based MASH CRN scoring: CNN pixel-level predictions for each histological feature (for example, fibrosis or steatosis) were clustered using GNN models and a score predicted based on the spatial organization of the cluster. To correct for pathologists’ bias, the GNN models were specified as ‘mixed effects’ models, biases were learned and the GNNs were deployed with predictions using only the unbiased estimate. GNN nodes and edges were built from CNN predictions of relevant histologic features derived from deployment of the H&E, MT and artifact models. **e**, Output: this two-stage ML approach produced patient-level predictions of MASH CRN MAS component scores and fibrosis stage.

**Fig. 2:**
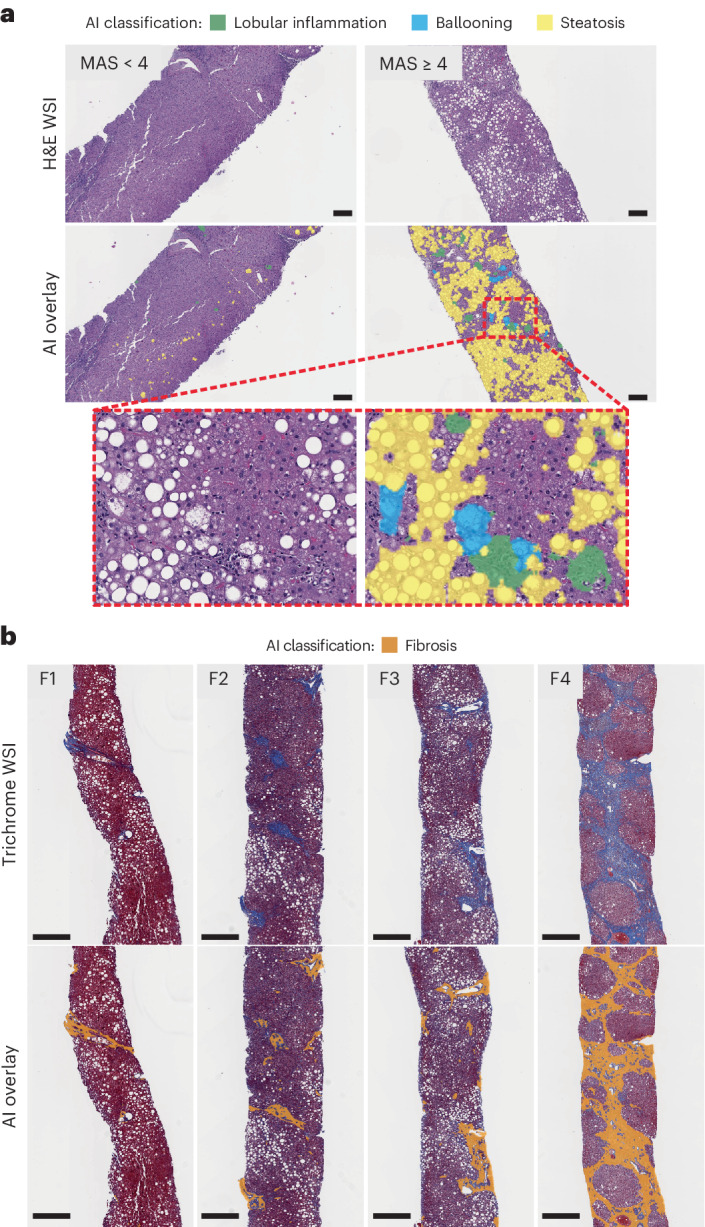
AI-based detection and scoring of MAS components and fibrosis. The MASH algorithm can detect histopathologic features on WSIs across a range of MASH disease severity. **a**, Representative H&E-stained slides show AI overlays highlighting regions of steatosis, lobular inflammation and ballooning. Representative cases corresponding to MAS < 4 (total *n* = 148) and MAS ≥ 4 (total *n* = 483), according to both pathologist consensus scoring and AI in the test set, are shown. The inset is a magnified field showing the presence of the three MAS components. Scale bar, 0.2 mm. **b**, Representative MT-stained slides of each MASH CRN fibrosis stage show AI-generated overlays highlighting regions of fibrosis present on biopsies. Representative cases corresponding to MASH CRN fibrosis stages F1 (total *n* = 159), F2 (total *n* = 146), F3 (total *n* = 278) and F4 (total *n* = 23), according to both pathologist consensus scoring and AI in the test set, are shown. These AI-generated overlays allow for qualitative review of model performance. Scale bar, 0.5 mm.

**Extended Data Fig. 1:**
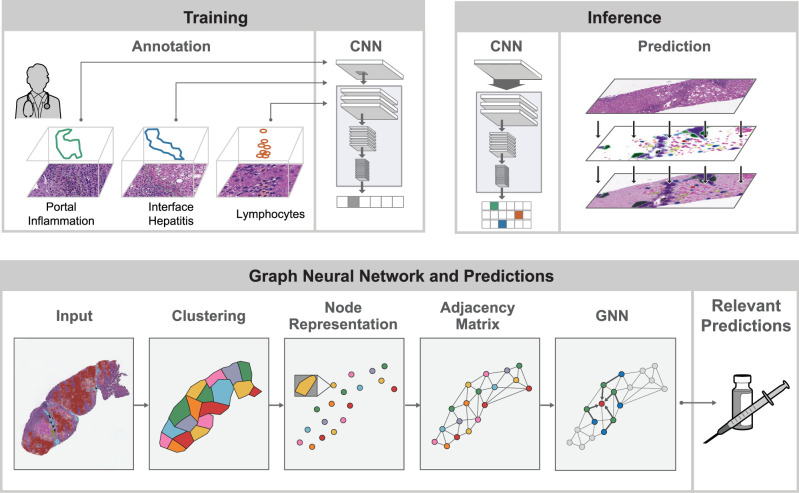
AIM-MASH CNN and GNN model training and predictions. CNN, convolutional neural network; GNN, graph neural network; AIM, Intelligence based Measurement; MASH, metabolic dysfunction-associated steatohepatitis.

**Extended Data Fig. 2:**
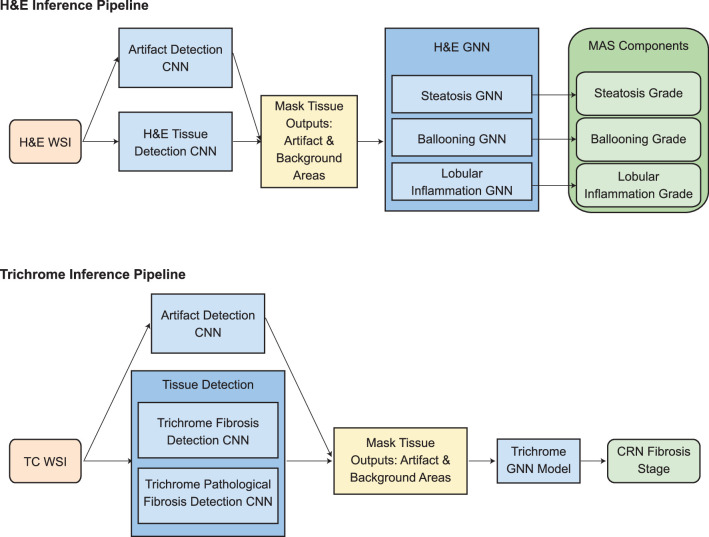
AIM-MASH H&E and Trichrome inference pipelines. H&E, hematoxylin and eosin; TC, trichrome.

**Extended Data Fig. 3:**
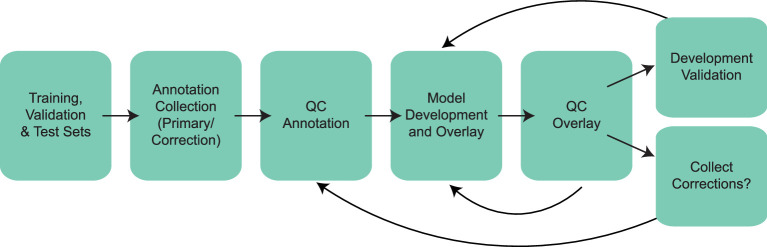
Segmentation model development process. QC, quality control.

AIM-MASH consists of multiple convolutional neural network (CNN) and graph neural network (GNN) models that each generate different categories of histologic readouts (Fig. 1 and Extended Data Figs. 1 and 2). CNN-based AI tissue, artifact and fibrosis models were trained using 103,579 pathologist-provided annotations (from 59 pathologists with expertise in MASH histology) of 8,747 hematoxylin and eosin (H&E) and 7,660 Masson’s trichrome (MT) WSIs from six completed phase 2b and phase 3 MASH clinical trials (Supplementary Table 1)^15–21^. These cohorts were split into training (∼70%), validation (∼15%) and test (∼15%) sets. Tissue, artifact and fibrosis models segmented relevant histological features (for example, metabolic dysfunction-associated steatotic liver disease activity score (MAS) components and fibrosis) to perform pixel-level mapping and slide-level feature quantification (Figs. 1 and 2). The overall segmentation model development process is shown in Extended Data Fig. 3.

GNN-based models received the CNN-derived outputs from the same dataset as inputs and were trained to predict MASH CRN ordinal grades or stages and corresponding continuous scores for each cardinal histologic feature of MASH^22^ (Figs. 1 and 2 and Extended Data Fig. 1).

## Model outputs

### Tissue overlays

Using WSIs of H&E- or MT-stained slides (Fig. 1a), a CNN-based artifact model was trained to distinguish evaluable liver tissue from tissue artifacts (for example, tissue folds, out-of-focus areas) and WSI background (Fig. 1b). H&E CNNs segmented MAS component features (macrovesicular steatosis, hepatocellular ballooning and lobular inflammation) and other relevant features, including portal inflammation, microvesicular steatosis, interface hepatitis and normal hepatocytes (that is, hepatocytes not exhibiting steatosis or ballooning). MT CNNs were trained to segment large intrahepatic septal and subcapsular regions (nonpathologic fibrosis), pathologic fibrosis and bile ducts (Fig. 1c). Model-derived predictions for location and distribution of each artifact and tissue feature were displayed as colorized overlays per WSI, enabling pathologists to review the model’s feature predictions for quality (Fig. 2).

### Histologic feature proportionate area measurements

CNN-derived histologic feature predictions were quantified to generate slide-level area measurements per feature. These measurements were expressed both as raw area quantities (mm^2^) and as percentages of relevant histology and artifact normalized relative to total usable (artifact-free) tissue area in the WSI. Artifact proportionate area measurements enabled efficient slide-level quality assessments and exclusion of inadequate image areas. Proportionate area measurements for H&E and MT MASH features, such as steatosis, ballooning, inflammation and fibrosis, were used to evaluate disease activity and severity.

### Model-derived predictions for MASH CRN grades and stages

**Extended Data Fig. 4:**
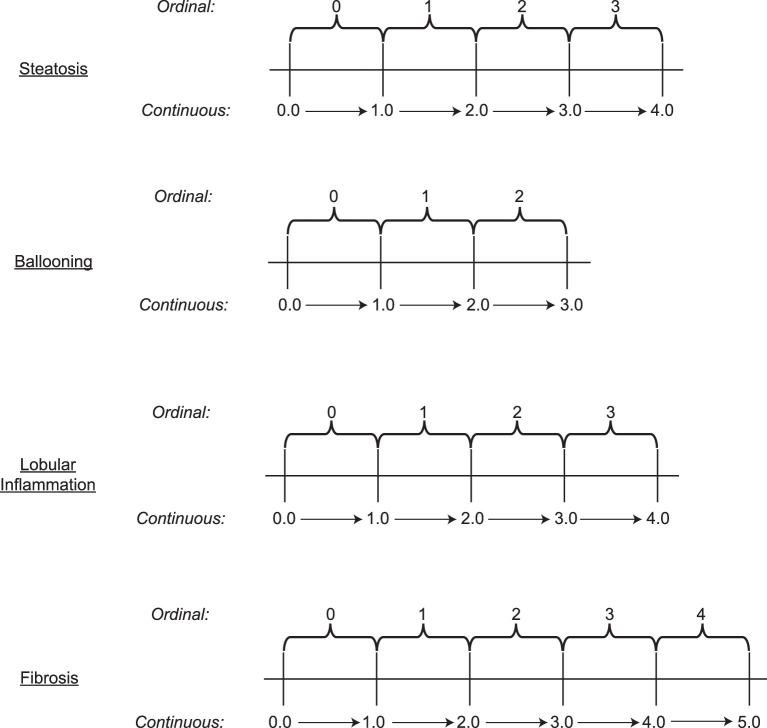
Mapping of continuous scores. Continuous MAS (steatosis, ballooning and lobular inflammation) and CRN fibrosis scores were produced by mapping GNN-derived ordinal grades/stages to bins, such that ordinal scores are spread over a continuous range (unit distance of 1). This continuous scoring system allows a more granular measurement of histological changes occurring at the subordinal level, while maintaining fidelity to the accepted ordinal scoring system.

Spatially resolved predictions from CNN image segmentation algorithms were used as inputs, and pathologists provided slide-level MASH CRN grades/stages as labels to train GNNs (Methods). GNNs were trained to predict MASH CRN steatosis grade, lobular inflammation grade and hepatocellular ballooning grade from H&E-stained WSIs, and fibrosis stage from MT-stained WSIs (Fig. 1d,e). To generate interpretable, high-resolution MASH CRN grades and stages, GNN-predicted scores calculated on a continuum were mapped to bins, each equivalent to one grade or stage (Extended Data Fig. 4). For example, the continuous range for MASH CRN steatosis grade 0 was 0–1, for grade 1 was 1–2, for grade 2 was 2–3 and for grade 3 was 3–4.

## Model performance repeatability and accuracy

In initial model performance testing relevant for application to both enrollment criteria and endpoints, AIM-MASH algorithm scoring was perfectly repeatable. For each of the four cardinal histologic features, a comparison of ten independent AIM-MASH reads per WSI resulted in a model versus model agreement rate of 100% (*κ* = 1; Supplementary Table 2), in contrast to previously reported intra-pathologist agreement using conventional approaches for consecutive reads, which was variable across features and ranged from 37% to 74% (ref. ^6^).

AIM-MASH performance accuracy was assessed using a mixed leave-one-out (MLOO) approach (Methods). Comparing model scoring predictions with a pathologist-based consensus for each of the four histologic features, model versus consensus agreement rates fell within the range of previously reported rates of inter-pathologist agreement (Table 1)^9,13^. The model versus consensus agreement rate was greatest for steatosis (*κ* = 0.74, 95% confidence interval (95% CI) 0.71–0.77), followed by ballooning (*κ* = 0.70, 95% CI 0.66–0.73), lobular inflammation (*κ* = 0.67, 95% CI 0.64–0.71) and fibrosis (*κ* = 0.62, 95% CI 0.58–0.65). In addition, agreement between the model and consensus was greater than agreement for any individual pathologist against the other three reads, and greater than any mean pairwise pathologist agreement (Table 1).

**Table 1:** Model performance accuracy assessment

Histologic feature	AIM-MASH versus consensus	Mean pathologist versus consensus	Mean pairwise pathologist agreement	Inter-reader concordance (*n* = 446)^13^
Lobular inflammation	0.67 (0.64–0.71)	0.64 (0.62–0.67)	0.58 (0.55–0.6)	0.46 (0.34–0.58)
Ballooning	0.70 (0.66–0.73)	0.66 (0.63–0.69)	0.61 (0.59–0.64)	0.54 (0.44–0.65)
Steatosis	0.74 (0.71–0.77)	0.69 (0.66–0.72)	0.62 (0.6–0.65)	0.77 (0.69–0.84)
Fibrosis	0.62 (0.58–0.65)	0.59 (0.57–0.62)	0.54 (0.51–0.56)	0.75 (0.67–0.82)

Data are presented as agreement rate (95% CI). AIM-MASH performance was tested on an external, held-out dataset comprising 640 H&E and 634 trichrome WSIs from EMMINENCE, a phase 2b MASH clinical trial. Agreement rates for AIM-MASH grades/stages versus a consensus of three expert pathologists were superior to mean agreement between any individual pathologist and a panel comprising the other two pathologists and the model, and superior to any mean pairwise pathologist agreement. *κ* statistics from Kleiner and colleagues^13^ are reported in the rightmost column as a comparison with recently published results from the pathology committee of the MASH CRN.

## Clinical utility of model-derived histology assessment

### AI-based evaluation of clinical trial enrollment criteria

For patients with noncirrhotic MASH and fibrosis, the FDA has proposed criteria for MASH clinical trial enrollment^23^. To demonstrate its clinical relevance, AIM-MASH was deployed on WSIs from two completed phase 2b MASH clinical trials^24,25^ to generate scores based on histologic criteria and identify patients eligible for enrollment (Supplementary Table 1, analytic performance test set). AI-derived predictions for each cohort were compared with each trial’s central pathologist (CP) scores of the same cohorts, as well as individual and consensus scores provided by three pathologists with expertise in MASH histology.

**Fig. 3:**
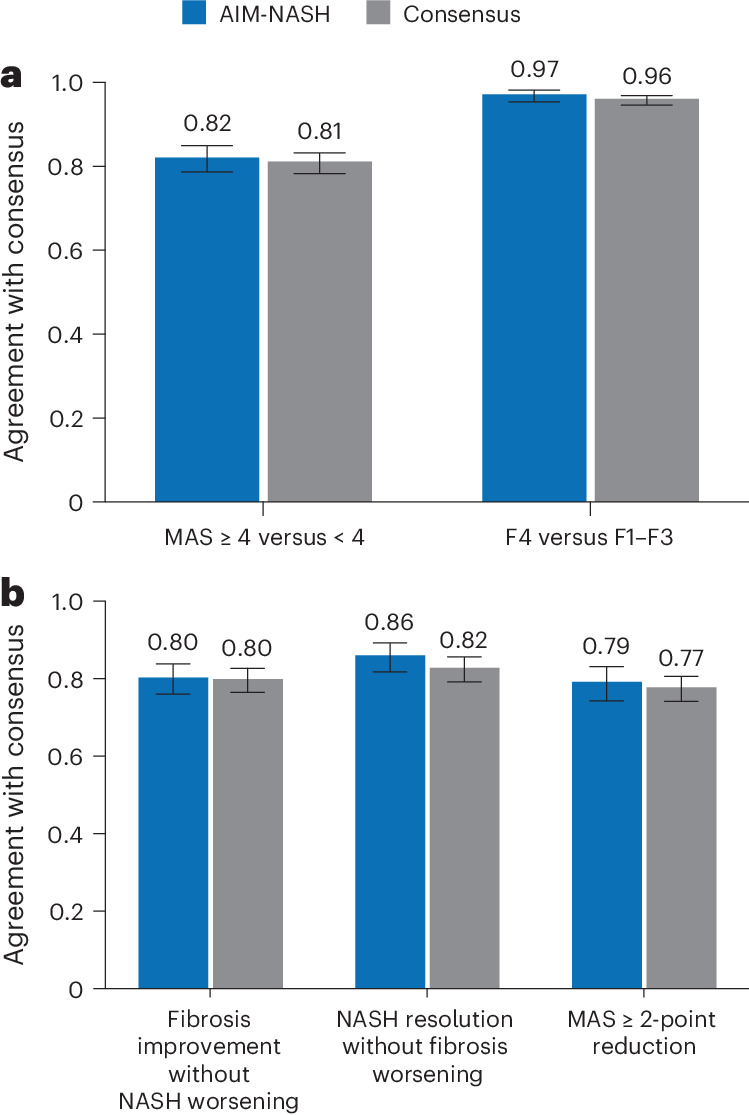
AI-based grading/staging of enrollment criteria and efficacy endpoints. **a**, Model-derived scores distinguished fibrosis stages F1–F3 versus F4 and MAS ≥ 4 (with each component grade ≥1) versus MAS < 4, criteria used to determine trial enrollment, using biopsies from the STELLAR-3 and STELLAR-4 clinical trials (*n* = 605). AIM-MASH agreement with consensus was comparable to that of each pathologist. Bar plots represent the point estimate of each enrollment criteria endpoint, and whiskers represent the 95% CIs estimated using 10,000 bootstrap samples. **b**, For assessment of efficacy endpoints commonly used in phase 2b and phase 3 MASH clinical trials, AIM-MASH agreement with consensus was comparable to that of an average pathologist. Assessment was performed on an external held-out validation dataset from a phase 2b MASH clinical trial using biopsies of patients meeting the following endpoints: fibrosis improvement without MASH worsening (*n* = 279), MASH resolution without fibrosis worsening (*n* = 279) and MAS reduction ≥2 (*n* = 326). Bar plots represent the point estimate of each enrollment criteria endpoint, and whiskers represent the 95% CIs estimated using 10,000 bootstrap samples.

Model-derived histologic predictions from 605 WSIs^24^ were used to calculate MASH CRN scores and distinguish MAS ≥ 4 (with each component grade ≥1) from MAS < 4, criteria used to determine trial enrollment (Supplementary Table 1). The AIM-MASH versus consensus percentage agreement (0.82, 95% CI 0.79–0.85) was comparable to that of an average pathologist versus consensus (0.81, 95% CI 0.78–0.83; Fig. 3a). A similar result was observed for fibrosis. For distinguishing fibrosis stages 1–3 (F1–F3) versus F4, the model versus consensus agreement was 0.97 (95% CI 0.95–0.98), similar to the average pathologist versus consensus agreement of 0.96 (95% CI 0.95–0.97; Fig. 3a).

### AI-based evaluation of clinical trial endpoints

For patients with noncirrhotic MASH and fibrosis, the FDA has proposed criteria for MASH clinical trial endpoint assessment^23^. Recommended MASH trial endpoints include evidence of efficacy using a histologic endpoint of MASH resolution or fibrosis improvement (late phase 2b trials), or both MASH resolution and fibrosis improvement for phase 3 trials.

Next, AIM-MASH predictions were used to determine component scores and evaluate composite endpoints in an exploratory retrospective analysis. AIM-MASH-derived histologic changes from baseline were compared with a consensus determination of the endpoints by three expert pathologists. Overall, AIM-MASH-based grading and staging for histologic endpoint assessment were comparable to those of mean individual pathologist versus consensus (Fig. 3b). For fibrosis improvement without worsening of MASH, both AIM-MASH versus consensus and pathologist versus consensus percentage agreement rates were 0.80 (95% CI 0.76–0.84 and 95% CI 0.77–0.83, respectively). For MASH resolution without worsening of fibrosis, model versus consensus agreement (0.86, 95% CI 0.82–0.89) was moderately greater than the pathologist versus consensus (0.82, 95% CI 0.79–0.86). A similar result was observed for a ≥2-point reduction in MAS, where the model versus consensus agreement (0.79, 95% CI 0.74–0.83) was comparable to the pathologist versus consensus agreement (0.77, 95% CI 0.74–0.81).

### AI-based detection of treatment response in clinical trials

Accurate assessment of treatment response is necessary for successful adoption of any new tool into MASH clinical trials. To demonstrate AIM-MASH’s ability to measure histologic response to a therapeutic, we performed a retrospective analysis of drug efficacy in the ATLAS phase 2b clinical trial (NCT03449446)^25^. ATLAS evaluated the efficacy of two drugs, cilofexor (CILO) and firsocostat (FIR), as monotherapies and in combination (CILO + FIR) in patients with advanced (F3–F4) fibrosis. Although no treatment arm achieved statistical significance for the primary endpoint, the cohort that received the combination of CILO + FIR showed the greatest improvement in histology relative to placebo^25^. AIM-MASH models were deployed on digitized WSIs (*n* = 99) from baseline and week 48 biopsies. Model predictions for ordinal MASH CRN grades/stages were generated and compared with CP measurements of grades/stages for the trial’s primary and two exploratory endpoints. In addition to computing the proportion of responders per endpoint, treatment arm and evaluation method, the difference in proportion of responders between CILO + FIR and placebo (placebo-adjusted response rate) was also computed.

**Fig. 4:**
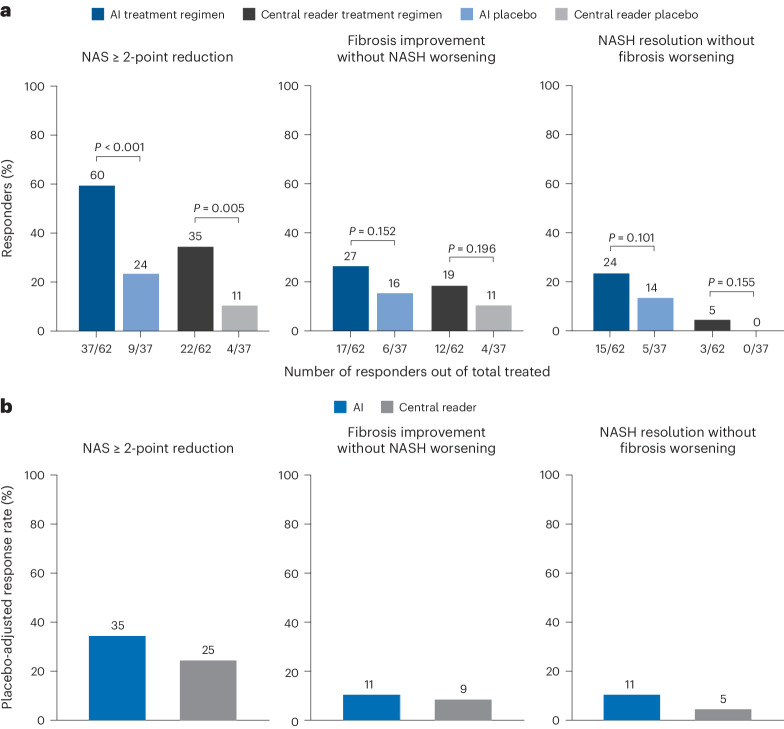
AIM-based retrospective drug efficacy assessment. AIM-MASH models were deployed on WSIs from baseline and week 48 biopsies from patients enrolled in the phase 2b ATLAS trial, which evaluated combination therapies for individuals with advanced MASH fibrosis. **a**, For the trial endpoints of MAS ≥ 2-point improvement, fibrosis improvement without worsening of MASH and MASH resolution without worsening of fibrosis, AIM-MASH models showed a greater proportion of responders compared with that determined by the trial central reader. For MAS ≥ 2-point improvement, odd ratios (ORs) for AI and central reader were 5.1 (95% CI 2.0–13.1) and 5.7 (95% CI 1.6–20.2), respectively; Cochran–Mantel–Haenszel (CMH) test statistics were 11.9 (*P* = 0.0006) and 7.9 (*P* = 0.005), respectively. For fibrosis improvement without worsening of MASH, ORs for AI and central reader were 2.2 (95% CI 0.7–6.3) and 2.2 (95% CI 0.6–7.7), respectively; CMH test statistics were 2.1 (*P* = 0.152) and 1.7 (*P* = 0.196), respectively. For MASH resolution without worsening of fibrosis, OR for AI was 2.7 (95% CI 0.8–8.8); OR for central reader was undefined, as no placebo responders were identified. CMH test statistics were 2.7 (*P* = 0.101) for AI and 2.0 (*P* = 0.155) for central reader. Sample sizes varied depending on data availability. **b**, The placebo-adjusted response rate detected by AIM-MASH was greater than that detected by the central reader.

AIM-MASH detected a greater proportion of treatment responders in the CILO + FIR group for all three endpoints measured compared with the CP (≥1-stage fibrosis improvement without MASH worsening, 27% versus 19%; MASH resolution without fibrosis worsening, 24% versus 5%; ≥2-point reduction in MAS, 60% versus 35%; Fig. 4a), in addition to showing a greater response in treated patients relative to placebo for all three endpoints (Fig. 4b).

## AI-enabled continuous scoring of MASH CRN components

As an initial exploration of alternative scoring systems to MASH CRN ordinal scoring, we developed a continuous scoring system that detects histologic changes that may occur within the range of an ordinal bin. The continuous system was mapped directly to the ordinal MASH CRN scoring system, facilitating interpretation and navigation between the ordinal and continuous systems when assessing therapeutic effect in MASH clinical trials (Extended Data Fig. 4).

### Biological relevance of continuous scoring

**Fig. 5:**
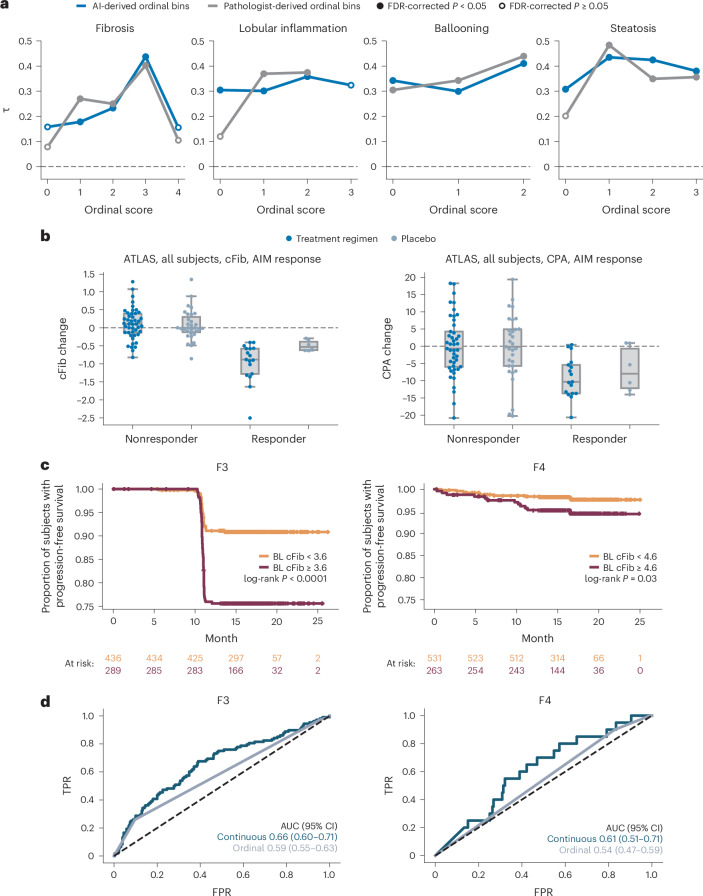
AI-based continuous MASH CRN scores. **a**, Correlation of AI-based continuous scores with mean scores across three pathologists from EMMINENCE in the analytic performance test set. Results are shown for both AI-derived ordinal bins (blue) and pathologist-derived ordinal bins (gray). Plotted values were derived from Kendall’s tau (τ) rank correlation analysis. FDR correction of *P* values was performed using the Benjamini–Hochberg procedure. Filled circles indicate statistical significance, FDR-corrected *P* < 0.05. **b**, cFib versus CPA measurements in primary endpoint responders in the ATLAS clinical trial. cFib and CPA were compared between patients receiving treatment and placebo using two-sided Mann–Whitney *U* tests. In primary endpoint responders, continuous fibrosis scores were significantly reduced in treated patients (*n* = 17) versus placebo patients (*n* = 6; Mann–Whitney *U* = 20.0, *P* = 0.02), while proportionate area fibrosis measurements were not significantly reduced (Mann–Whitney *U* = 39.0, *P* = 0.21). cFib and CPA values for patients classified as nonresponders (*n* = 76), in the treatment (*n* = 45) or placebo (*n* = 31) group, are also shown. Boxes represent the 25th percentile, median and 75th percentile of the data. Whiskers extend to points that lie within 1.5-fold of the inter-quartile range of the 25th and 75th percentiles. **c**, Stratifi**c**ation of patients with BL F3 or F4 fibrosis from STELLAR-3 and STELLAR-4 trial cohorts into rapid (red) and slow (orange) progressors based on continuous score cutoffs of 3.6 and 4.6, respectively. Kaplan–Meier and Cox proportional hazards regression analyses are shown. F3: log-rank statistic = 31.0, *P* = 2.6 × 10^−8^; F4: log-rank statistic = 4.8, *P* = 0.028. Rounded cutoffs were chosen to maximize hazards. **d**, Discriminatory accuracy of AI-derived continuous scores versus ordinal scores to predict progression to cirrhosis (left) and LRE (right) in STELLAR-3 and STELLAR-4 trial cohorts. In both cases, using receiver operating characteristic analysis, the continuous AUC was significantly greater (progression to cirrhosis: 0.66 (95% CI 0.60–0.71) versus 0.59 (95% CI 0.55–0.60); progression to LRE: 0.61 (95% CI 0.51–0.71) versus 0.54 (95% CI 0.47–59)). AUC, area under the receiver operating characteristic curve; BL, baseline; FDR, false discovery rate; FPR, false positive rate; τ, Kendall’s rank correlation coefficient for ordinal scores; TPR, true positive rate.

AI-enabled continuous scoring was evaluated by correlating continuous scores against mean scores from three pathologists in a held-out dataset (640 H&E and 634 trichrome WSIs)^24^. Continuous scores significantly correlated with mean pathologist scores, confirming alignment between machine learning (ML)-derived continuous scores and directional bias of panel-based pathologist scoring (Fig. 5a). These results suggest that the disease severity was similarly captured through subordinal measurements both by AIM-MASH and by the panel of pathologists but could not be captured by a single pathologist providing ordinal scores for staging and grading (Fig. 5a).

To further cross-validate the AI-derived continuous MASH CRN scores with other lines of clinical evidence, continuous scores were correlated with corresponding noninvasive test (NIT) metrics in the ATLAS dataset^25^. NITs that correlate strongly with specific histologic features or that were developed to serve as biomarkers for these features were correlated with the relevant continuous MASH CRN grades/stages (Supplementary Table 3). AI-derived continuous fibrosis stage was significantly correlated with liver stiffness by FibroScan (*τ*: 0.33, *P* < 0.001), Fibrosis-4 (*τ*: 0.23, *P* < 0.001), enhanced liver fibrosis test (*τ*: 0.22, *P* < 0.001), tissue inhibitor of metalloproteinases 1 (*τ*: 0.11, *P* = 0.02) and amino terminal propeptide of type III procollagen (*τ*: 0.14, *P* < 0.01); continuous steatosis grade was not significantly correlated with the same NIT measures. Similarly, whereas continuous steatosis grade was significantly correlated with magnetic resonance imaging–proton density fat fraction (*τ*: 0.52, *P* < 0 .001), continuous fibrosis stage was not correlated with magnetic resonance imaging–proton density fat fraction (*τ*: −0.11, *P* = 0.24). Continuous lobular inflammation grade was significantly correlated with C-reactive protein (*τ*: 0.13, *P* < 0.01) and adiponectin levels (*τ*: –0.15, *P* < 0.01), while continuous ballooning grade was significantly correlated with glycated hemoglobin (*τ*: 0.16, *P* < 0.001). Notably, both continuous fibrosis stage and continuous steatosis grade were significantly correlated with collagen proportionate area (CPA) by morphometry, but in opposite directions (continuous fibrosis stage: *τ*: 0.56, *P* < 0.001; continuous steatosis grade: *τ*: −0.16, *P* < 0.001), consistent with previously reported reductions in steatosis with progression of fibrosis in MASH^26–29^.

### AI-derived continuous MASH CRN fibrosis staging

To assess the relative sensitivities of AI-based continuous MASH CRN fibrosis staging and conventional continuous measures, AIM-MASH ordinal fibrosis scores and AI-derived proportionate area of fibrosis measurement (surrogate for CPA) in MT images from ATLAS^25^ were computed in CILO + FIR- and placebo-treated cohorts. Continuous AIM-MASH fibrosis staging (cFib) captures greater changes in treatment versus placebo over conventional continuous fibrosis measures. In primary endpoint responders, treated patients showed a significantly greater reduction in cFib than placebo patients (Mann–Whitney *U* = 20.0, *P* = 0.02). Proportionate area of fibrosis was not significantly reduced in treated patients relative to placebo (Mann–Whitney *U* = 39.0, *P* = 0.21; Fig. 5b). In addition, cFib scores increased in nonresponders but decreased in responders, showing that continuous scoring was able to identify worsening fibrosis in patients not responding to treatment (Fig. 5b).

### Advantage of continuous scoring for predicting outcomes

To assess the potential utility of the continuous scoring approach for patient stratification and for predicting outcomes, we examined the prognostic utility of continuous scoring for predicting progression to cirrhosis (F4) in patients with bridging (F3) fibrosis at baseline or predicting liver-related events (LREs) in patients with cirrhosis at baseline in the STELLAR-3 (NCT03053050) and STELLAR-4 (NCT03053063) MASH clinical trial cohorts^15^, respectively. Associations between continuous scores at baseline and clinical disease progression through the end of follow-up were determined using the Kaplan–Meier method and Cox proportional hazards regression analysis, with rounded cutoffs selected to maximize hazards. cFib cutoffs of 3.6 and 4.6 maximized the stratification of patients into slow versus rapid progressors to cirrhosis or LREs, respectively (Fig. 5c). AI-derived continuous scoring showed higher discriminatory accuracy for predicting progression to cirrhosis and LREs than ML-derived ordinal scoring (Fig. 5d).

## Discussion

Pathologist assessment of liver histopathology is central to the evaluation of disease severity and serves as the basis for patient selection and treatment efficacy assessment in MASH clinical trials. Histologic evaluation in MASH clinical trials has been limited by intra- and inter-pathologist variability in histologic grading and staging^6,30^. However, despite the FDA and MASH CRN having proposed panel scoring, these guidelines have yet to be standardized or widely adopted, and variability in assessment, even among expert pathologists, remains high^6,12^.

To assist pathologists in locating and evaluating critical histologic signatures of MASH disease progression and regression, we developed AIM-MASH, a suite of algorithms that reproducibly predict the location, extent and severity of histologic biomarkers of MASH via both AI-derived recapitulation of MASH CRN ordinal grading and staging and AI-based qualitative and quantitative metrics. These algorithms also enable reproducible scoring and consistent measurement of changes in disease severity between baseline and end of treatment in MASH clinical trials, harmonizing with the histologic endpoints recommended by both the FDA and EMA. Integrating AI-based digital pathology tools such as AIM-MASH into MASH clinical trial workflows using validated WSI viewing platforms^31^ has the potential to positively impact the development of MASH therapeutics by ensuring consistent and reproducible pathologist assessments, resulting in improved identification of patients with MASH for trial enrollment, more robust measurement of histologic endpoints and greater sensitivity to drug effects, increasing clinical trial success and improving patient outcomes.

Here, we demonstrate consistently accurate and reproducible AIM-MASH scoring. AI-derived predictions for MASH CRN steatosis grade, lobular inflammation grade, ballooning grade and fibrosis stage were concordant with expert pathologists’ consensus MASH CRN grading/staging in a MASH clinical trial. AIM-MASH performance was tested by treating the model as an independent reader within a panel. Our results provide evidence that the model did not internalize any individual pathologist’s scoring biases, but instead learned to grade and stage histologic features in alignment with a consensus of pathologists with expertise in MASH. These results suggest that AIM-MASH captured features and changes in histology over time and in response to drug treatment in an unbiased manner that aligned with expert pathologist interpretation. Model-derived ordinal scores recapitulated patient enrollment and endpoint measurement in a completed phase 2b MASH clinical trial and were 100% reproducible when the analysis was repeated on the same images, suggesting that this approach could enable consistent measurement of disease severity within and across timepoints and clinical trials. Further analytical validation will assess reproducibility with various pre-analytic factors (including different scanners, drug candidates, screening and enrolled populations, stain quality, biopsy and section quality)^32^. The accuracy and reproducibility of AIM-MASH may prove especially valuable in assisting pathologists to achieve reproducible results for cases in which the histopathology is borderline between two grades/stages and discordance among pathologists is common.

Reproducible and accurate AIM-MASH grading and staging of histologic features can detect response to drug treatment with comparable accuracy to pathologists with expertise in assessment of MASH. Retrospective assessment of primary and exploratory endpoints of the EMMINENCE (NCT02784444)^24^ and phase 2b ATLAS clinical trials^25^ showed that AIM-MASH achieved a high level of scoring accuracy and superior reproducibility compared with pathologists. In addition, AIM-MASH detected a greater proportion of responders in treated patients than manual scoring even when adjusting for the proportion of placebo patient responders. This trend has been observed in retrospective analyses of other trial cohorts (Supplementary Table 4)^33–36^. In one case, AIM-MASH revealed statistically significant differences in response rates between treated and placebo patients in contrast to manual assessment^35^. AIM-MASH may have future applicability in clinical trials to determine disease severity and sensitive assessment of drug efficacy. Validation studies required to support AIM-MASH application to prospective clinical trial workflows are underway, including rigorous analytical validation to verify repeatability and reproducibility across scanners and scanner operators, and clinical validation to verify the efficacy, utility and scalability of an AI-assisted clinical trial workflow across multiple clinical trial datasets^32^.

Achieving the surrogate biopsy-based endpoints recommended by regulatory bodies for MASH clinical trials has been exceedingly difficult, in part owing to the slow rate at which MASH progresses and regresses^37^. To identify alternative biopsy-based biomarkers to understand MASH pathogenesis and monitor disease activity, we previously used AI models to investigate non-CRN histological features that are associated with clinical outcomes and may be predictive of risk of disease progression in STELLAR-3, STELLAR-4 and ATLAS. For example, we have shown that the area of portal inflammation was predictive of risk of disease progression, LREs and cirrhosis^5,38^, and was one of three significant model-predicted human interpretable features (along with the area of bile duct/ductules and fibrosis) used to identify a gene signature predictive of risk of clinical events^39^. We also previously showed that the area of bile duct/ductules measured by AI was associated with a higher risk of LREs^38^. Another notable human interpretable feature identified using our AI-based measurements was the ratio of steatosis to hepatocellular ballooning, where patients with higher hepatocellular ballooning to steatosis were more likely to experience clinical events^5^. To address the challenge of the slow pace of MASH disease progression and regression, several measurement systems that detect subordinal levels of histologic change have been proposed^40^, including the utility of AI-based continuous measures of fibrosis for detecting subtle, yet statistically significant, changes in fibrosis in response to treatment^5^.

The continuous scoring system we present here maps each MASH CRN grade/stage to a bin derived from the ordinal scoring system, allowing direct comparison between the ordinal and continuous scoring systems. The AI-derived continuous MASH CRN scores strongly correlated with mean scores derived by a panel of expert pathologists. The directional bias of the panel was clearly reflected in the continuous scores and between these scores and relevant noninvasive MASH biomarkers that are known to correlate with specific histologic features and clinical outcomes^27^. The AIM-MASH-based continuous MASH CRN fibrosis score was more sensitive to treatment-induced changes in fibrosis than the gold standard continuous CPA and was more strongly predictive of progression to cirrhosis and liver-related complications than AI-based ordinal MASH CRN grades/stages. Additionally, the continuous fibrosis score enabled the definition of cutoffs that stratified patients with MASH with stage 3 (F3) or stage 4 (F4) fibrosis into slow versus rapid progressors. These results suggest several important applications for continuous histologic scoring in MASH in both translational and clinical settings.

Although continuous scoring may offer a means to measure subtle changes in MASH that are more realistically achievable on the timescale of clinical trials, a limitation is that it presents disease progression and regression on a linear scale, which is inconsistent with how MASH progresses and regresses^37,41^: for instance, a change in continuous fibrosis stage from 3.0 to 3.2 may reflect a different amount of change in disease severity than a change of similar magnitude (for example, from 4.0 to 4.2). Future experiments should investigate whether mapping a nonlinear system to a linear scale complicates measurement of changes in disease severity in response to treatment and whether a scale that more closely approximates the manner in which MASH disease progresses and regresses is feasible. Furthermore, clinically meaningful thresholds of continuous scoring are not yet known, and changes in the continuous fibrosis score must be defined and characterized to determine whether a sub-integer reduction in fibrosis score is associated with improved clinical outcome before this system can be widely adopted.

The results presented here highlight how collaboration between AI developers and pathologists with expertise in MASH can make consequential steps toward solving the problems inherent to MASH histologic assessment that lead to the failure of clinical trials. To this end, AIM-MASH is being evaluated by both the FDA and the EMA for qualification as a drug discovery tool for use in clinical trials. This tool is well poised to improve the accuracy and reproducibility of pathologists’ evaluation of liver biopsies within scalable workflows that can accommodate the increasing demand for MASH clinical trials. With the urgent unmet need of patients with MASH, we hope that AIM-MASH can aid pathologists in the clinical trial setting. AIM-MASH also has potential as a research use only tool to investigate histological features such as portal inflammation as new biomarkers or scoring systems, such as our continuous scores, in clinical cohorts.

## Methods

### Compliance

AI-based computational pathology models and platforms to support model functionality were developed using Good Clinical Practice/Good Clinical Laboratory Practice principles, including controlled process and testing documentation.

### Ethics

This study was conducted in accordance with the Declaration of Helsinki and Good Clinical Practice guidelines. Anonymized liver tissue samples and digitized WSIs of H&E- and trichrome-stained liver biopsies were obtained from adult patients with MASH that had participated in any of the following complete randomized controlled trials of MASH therapeutics: NCT03053050 (ref. ^15^), NCT03053063 (ref. ^15^), NCT01672866 (ref. ^16^), NCT01672879 (ref. ^17^), NCT02466516 (ref. ^18^), NCT03551522 (ref. ^21^), NCT00117676 (ref. ^19^), NCT00116805 (ref. ^19^), NCT01672853 (ref. ^20^), NCT02784444 (ref. ^24^), NCT03449446 (ref. ^25^). Approval by central institutional review boards was previously described^15–21,24,25^. All patients had provided informed consent for future research and tissue histology as previously described^15–21,24,25^.

### Data collection

#### Datasets

ML model development and external, held-out test sets are summarized in Supplementary Table 1. ML models for segmenting and grading/staging MASH histologic features were trained using 8,747 H&E and 7,660 MT WSIs from six completed phase 2b and phase 3 MASH clinical trials, covering a range of drug classes, trial enrollment criteria and patient statuses (screen fail versus enrolled) (Supplementary Table 1)^15–21^. Samples were collected and processed according to the protocols of their respective trials and were scanned on Leica Aperio AT2 or Scanscope V1 scanners at either ×20 or ×40 magnification. H&E and MT liver biopsy WSIs from primary sclerosing cholangitis and chronic hepatitis B infection were also included in model training. The latter dataset enabled the models to learn to distinguish between histologic features that may visually appear to be similar but are not as frequently present in MASH (for example, interface hepatitis)^42^ in addition to enabling coverage of a wider range of disease severity than is typically enrolled in MASH clinical trials.

Model performance repeatability assessments and accuracy verification were conducted in an external, held-out validation dataset (analytic performance test set) comprising WSIs of baseline and end-of-treatment (EOT) biopsies from a completed phase 2b MASH clinical trial (Supplementary Table 1)^24,25^. The clinical trial methodology and results have been described previously^24^. Digitized WSIs were reviewed for CRN grading and staging by the clinical trial’s three CPs, who have extensive experience evaluating MASH histology in pivotal phase 2 clinical trials and in the MASH CRN and European MASH pathology communities^6^. Images for which CP scores were not available were excluded from the model performance accuracy analysis. Median scores of the three pathologists were computed for all WSIs and used as a reference for AI model performance. Importantly, this dataset was not used for model development and thus served as a robust external validation dataset against which model performance could be fairly tested.

The clinical utility of model-derived features was assessed by generated ordinal and continuous ML features in WSIs from four completed MASH clinical trials: 1,882 baseline and EOT WSIs from 395 patients enrolled in the ATLAS phase 2b clinical trial^25^, 1,519 baseline WSIs from patients enrolled in the STELLAR-3 (*n* = 725 patients) and STELLAR-4 (*n* = 794 patients) clinical trials^15^, and 640 H&E and 634 trichrome WSIs (combined baseline and EOT) from the EMINENCE trial^24^. Dataset characteristics for these trials have been published previously^15,24,25^.

#### Pathologists

Board-certified pathologists with experience in evaluating MASH histology assisted in the development of the present MASH AI algorithms by providing (1) hand-drawn annotations of key histologic features for training image segmentation models (see the section ‘Annotations’ and Supplementary Table 5); (2) slide-level MASH CRN steatosis grades, ballooning grades, lobular inflammation grades and fibrosis stages for training the AI scoring models (see the section ‘Model development’); or (3) both. Pathologists who provided slide-level MASH CRN grades/stages for model development were required to pass a proficiency examination, in which they were asked to provide MASH CRN grades/stages for 20 MASH cases, and their scores were compared with a consensus median provided by three MASH CRN pathologists. Agreement statistics were reviewed by a PathAI pathologist with expertise in MASH and leveraged to select pathologists for assisting in model development. In total, 59 pathologists provided feature annotations for model training; five pathologists provided slide-level MASH CRN grades/stages (see the section ‘Annotations’).

#### Annotations

##### Tissue feature annotations

Pathologists provided pixel-level annotations on WSIs using a proprietary digital WSI viewer interface. Pathologists were specifically instructed to draw, or ‘annotate’, over the H&E and MT WSIs to collect many examples of substances relevant to MASH, in addition to examples of artifact and background. Instructions provided to pathologists for select histologic substances are included in Supplementary Table 4 (refs. ^33–36^). In total, 103,579 feature annotations were collected to train the ML models to detect and quantify features relevant to image/tissue artifact, foreground versus background separation and MASH histology.

##### Slide-level MASH CRN grading and staging

All pathologists who provided slide-level MASH CRN grades/stages received and were asked to evaluate histologic features according to the MAS and CRN fibrosis staging rubrics developed by Kleiner et al.^9^. All cases were reviewed and scored using the aforementioned WSI viewer.

### Model development

#### Dataset splitting

The model development dataset described above was split into training (~70%), validation (~15%) and held-out test (∼15%) sets. The dataset was split at the patient level, with all WSIs from the same patient allocated to the same development set. Sets were also balanced for key MASH disease severity metrics, such as MASH CRN steatosis grade, ballooning grade, lobular inflammation grade and fibrosis stage, to the greatest extent possible. The balancing step was occasionally challenging because of the MASH clinical trial enrollment criteria, which restricted the patient population to those fitting within specific ranges of the disease severity spectrum. The held-out test set contains a dataset from an independent clinical trial to ensure algorithm performance is meeting acceptance criteria on a completely held-out patient cohort in an independent clinical trial and avoiding any test data leakage^43^.

#### CNNs

The present AI MASH algorithms were trained using the three categories of tissue compartment segmentation models described below. Summaries of each model and their respective objectives are included in Supplementary Table 6, and detailed descriptions of each model’s purpose, input and output, as well as training parameters, can be found in Supplementary Tables 7–9. For all CNNs, cloud-computing infrastructure allowed massively parallel patch-wise inference to be efficiently and exhaustively performed on every tissue-containing region of a WSI, with a spatial precision of 4–8 pixels.

##### Artifact segmentation model

A CNN was trained to differentiate (1) evaluable liver tissue from WSI background and (2) evaluable tissue from artifacts introduced via tissue preparation (for example, tissue folds) or slide scanning (for example, out-of-focus regions). A single CNN for artifact/background detection and segmentation was developed for both H&E and MT stains (Fig. 1).

##### H&E segmentation model

For H&E WSIs, a CNN was trained to segment both the cardinal MASH H&E histologic features (macrovesicular steatosis, hepatocellular ballooning, lobular inflammation) and other relevant features, including portal inflammation, microvesicular steatosis, interface hepatitis and normal hepatocytes (that is, hepatocytes not exhibiting steatosis or ballooning; Fig. 1).

##### MT segmentation models

For MT WSIs, CNNs were trained to segment large intrahepatic septal and subcapsular regions (comprising nonpathologic fibrosis), pathologic fibrosis, bile ducts and blood vessels (Fig. 1). All three segmentation models were trained utilizing an iterative model development process, schematized in Extended Data Fig. 2. First, the training set of WSIs was shared with a select team of pathologists with expertise in assessment of MASH histology who were instructed to annotate over the H&E and MT WSIs, as described above. This first set of annotations is referred to as ‘primary annotations’. Once collected, primary annotations were reviewed by internal pathologists, who removed annotations from pathologists who had misunderstood instructions or otherwise provided inappropriate annotations. The final subset of primary annotations was used to train the first iteration of all three segmentation models described above, and segmentation overlays (Fig. 2) were generated. Internal pathologists then reviewed the model-derived segmentation overlays, identifying areas of model failure and requesting correction annotations for substances for which the model was performing poorly. At this stage, the trained CNN models were also deployed on the validation set of images to quantitatively evaluate the model’s performance on collected annotations. After identifying areas for performance improvement, correction annotations were collected from expert pathologists to provide further improved examples of MASH histologic features to the model. Model training was monitored, and hyperparameters were adjusted based on the model’s performance on pathologist annotations from the held-out validation set until convergence was achieved and pathologists confirmed qualitatively that model performance was strong.

The artifact, H&E tissue and MT tissue CNNs were trained using pathologist annotations comprising 8–12 blocks of compound layers with a topology inspired by residual networks and inception networks with a softmax loss^44–46^. A pipeline of image augmentations was used during training for all CNN segmentation models. CNN models’ learning was augmented using distributionally robust optimization^47,48^ to achieve model generalization across multiple clinical and research contexts and augmentations. For each training patch, augmentations were uniformly sampled from the following options and applied to the input patch, forming training examples. The augmentations included random crops (within padding of 5 pixels), random rotation (≤360°), color perturbations (hue, saturation and brightness) and random noise addition (Gaussian, binary-uniform). Input- and feature-level mix-up^49,50^ was also employed (as a regularization technique to further increase model robustness). After application of augmentations, images were zero-mean normalized. Specifically, zero-mean normalization is applied to the color channels of the image, transforming the input RGB image with range [0–255] to BGR with range [−128–127]. This transformation is a fixed reordering of the channels and subtraction of a constant (−128), and requires no parameters to be estimated. This normalization is also applied identically to training and test images.

### GNNs

CNN model predictions were used in combination with MASH CRN scores from eight pathologists to train GNNs to predict ordinal MASH CRN grades for steatosis, lobular inflammation, ballooning and fibrosis. GNN methodology was leveraged for the present development effort because it is well suited to data types that can be modeled by a graph structure, such as human tissues that are organized into structural topologies, including fibrosis architecture^51^. Here, the CNN predictions (WSI overlays) of relevant histologic features were clustered into ‘superpixels’ to construct the nodes in the graph, reducing hundreds of thousands of pixel-level predictions into thousands of superpixel clusters. WSI regions predicted as background or artifact were excluded during clustering. Directed edges were placed between each node and its five nearest neighboring nodes (via the *k*-nearest neighbor algorithm). Each graph node was represented by three classes of features generated from previously trained CNN predictions predefined as biological classes of known clinical relevance. Spatial features included the mean and standard deviation of (*x*, *y*) coordinates. Topological features included area, perimeter and convexity of the cluster. Logit-related features included the mean and standard deviation of logits for each of the classes of CNN-generated overlays. Scores from multiple pathologists were used independently during training without taking consensus, and consensus (*n* = 3) scores were used for evaluating model performance on validation data. Leveraging scores from multiple pathologists reduced the potential impact of scoring variability and bias associated with a single reader.

To further account for systemic bias, whereby some pathologists may consistently overestimate patient disease severity while others underestimate it, we specified the GNN model as a ‘mixed effects’ model. Each pathologist’s policy was specified in this model by a set of bias parameters learned during training and discarded at test time. Briefly, to learn these biases, we trained the model on all unique label–graph pairs, where the label was represented by a score and a variable that indicated which pathologist in the training set generated this score. The model then selected the specified pathologist bias parameter and added it to the unbiased estimate of the patient’s disease state. During training, these biases were updated via backpropagation only on WSIs scored by the corresponding pathologists. When the GNNs were deployed, the labels were produced using only the unbiased estimate.

In contrast to our previous work, in which models were trained on scores from a single pathologist^5^, GNNs in this study were trained using MASH CRN scores from eight pathologists with experience in evaluating MASH histology on a subset of the data used for image segmentation model training (Supplementary Table 1). The GNN nodes and edges were built from CNN predictions of relevant histologic features in the first model training stage. This tiered approach improved upon our previous work, in which separate models were trained for slide-level scoring and histologic feature quantification. Here, ordinal scores were constructed directly from the CNN-labeled WSIs.

### GNN-derived continuous score generation

Continuous MAS and CRN fibrosis scores were produced by mapping GNN-derived ordinal grades/stages to bins, such that ordinal scores were spread over a continuous range spanning a unit distance of 1 (Extended Data Fig. 2). Activation layer output logits were extracted from the GNN ordinal scoring model pipeline and averaged. The GNN learned inter-bin cutoffs during training, and piecewise linear mapping was performed per logit ordinal bin from the logits to binned continuous scores using the logit-valued cutoffs to separate bins. Bins on either end of the disease severity continuum per histologic feature have long-tailed distributions that are not penalized during training. To ensure balanced linear mapping of these outer bins, logit values in the first and last bins were restricted to minimum and maximum values, respectively, during a post-processing step. These values were defined by outer-edge cutoffs chosen to maximize the uniformity of logit value distributions across training data. GNN continuous feature training and ordinal mapping were performed for each MASH CRN and MAS component fibrosis separately.

### Quality control measures

Several quality control measures were implemented to ensure model learning from high-quality data: (1) PathAI liver pathologists evaluated all annotators for annotation/scoring performance at project initiation; (2) PathAI pathologists performed quality control review on all annotations collected throughout model training; following review, annotations deemed to be of high quality by PathAI pathologists were used for model training, while all other annotations were excluded from model development; (3) PathAI pathologists performed slide-level review of the model’s performance after every iteration of model training, providing specific qualitative feedback on areas of strength/weakness after each iteration; (4) model performance was characterized at the patch and slide levels in an internal (held-out) test set; (5) model performance was compared against pathologist consensus scoring in an entirely held-out test set, which contained images that were out of distribution relative to images from which the model had learned during development.

### Statistical analysis

#### Model performance repeatability

Repeatability of AI-based scoring (intra-method variability) was assessed by deploying the present AI algorithms on the same held-out analytic performance test set ten times and computing percentage positive agreement across the ten reads by the model.

#### Model performance accuracy

To verify model performance accuracy, model-derived predictions for ordinal MASH CRN steatosis grade, ballooning grade, lobular inflammation grade and fibrosis stage were compared with median consensus grades/stages provided by a panel of three expert pathologists who had evaluated MASH biopsies in a recently completed phase 2b MASH clinical trial (Supplementary Table 1). Importantly, images from this clinical trial were not included in model training and served as an external, held-out test set for model performance evaluation. Alignment between model predictions and pathologist consensus was measured via agreement rates, reflecting the proportion of positive agreements between the model and consensus.

We also evaluated the performance of each expert reader against a consensus to provide a benchmark for algorithm performance. For this MLOO analysis, the model was considered a fourth ‘reader’, and a consensus, determined from the model-derived score and that of two pathologists, was used to evaluate the performance of the third pathologist left out of the consensus. The average individual pathologist versus consensus agreement rate was computed per histologic feature as a reference for model versus consensus per feature. Confidence intervals were computed using bootstrapping. Concordance was assessed for scoring of steatosis, lobular inflammation, hepatocellular ballooning and fibrosis using the MASH CRN system.

#### AI-based assessment of clinical trial enrollment criteria and endpoints

The analytic performance test set (Supplementary Table 1) was leveraged to assess the AI’s ability to recapitulate MASH clinical trial enrollment criteria and efficacy endpoints. Baseline and EOT biopsies across treatment arms were grouped, and efficacy endpoints were computed using each study patient’s paired baseline and EOT biopsies. For all endpoints, the statistical method used to compare treatment with placebo was a Cochran–Mantel–Haenszel test, and *P* values were based on response stratified by diabetes status and cirrhosis at baseline (by manual assessment). Concordance was assessed with κ statistics, and accuracy was evaluated by computing F1 scores. A consensus determination (*n* = 3 expert pathologists) of enrollment criteria and efficacy served as a reference for evaluating AI concordance and accuracy. To evaluate the concordance and accuracy of each of the three pathologists, AI was treated as an independent, fourth ‘reader’, and consensus determinations were composed of the AIM and two pathologists for evaluating the third pathologist not included in the consensus. This MLOO approach was followed to evaluate the performance of each pathologist against a consensus determination.

#### Continuous score interpretability

To demonstrate interpretability of the continuous scoring system, we first generated MASH CRN continuous scores in WSIs from a completed phase 2b MASH clinical trial (Supplementary Table 1, analytic performance test set). The continuous scores across all four histologic features were then compared with the mean pathologist scores from the three study central readers, using Kendall rank correlation. The goal in measuring the mean pathologist score was to capture the directional bias of this panel per feature and verify whether the AI-derived continuous score reflected the same directional bias.

### Reporting summary

Further information on research design is available in the Nature Portfolio Reporting Summary linked to this article.

## Online content

Any methods, additional references, Nature Portfolio reporting summaries, source data, extended data, supplementary information, acknowledgements, peer review information; details of author contributions and competing interests; and statements of data and code availability are available at 10.1038/s41591-024-03172-7.

## Supplementary information

Supplementary Information Supplementary Tables 1–9.

Reporting Summary

## Data Availability

The histopathology data collected for this study are maintained by PathAI to preserve patient confidentiality and the proprietary image analysis. Access to histopathology features will be granted to academic investigators without relevant conflicts of interest for noncommercial use who agree not to distribute the data. Access requests can be made to Andrew Beck (andy.beck@pathai.com). Any additional information required to reanalyze the data reported in this paper relating directly to the clinical datasets (STELLAR-3, STELLAR-4, GS-US-321-0105, GS-US-321-0106, GS‐US‐384‐1497, ENHANCE, HBV, PSC, EMMINENCE and ATLAS datasets) will be considered at the discretion of the source institute for the clinical trial in question. Requests will be considered from academic investigators without relevant conflicts of interest for noncommercial use who agree not to distribute the data. Data requests should be sent to Andrew Beck (andy.beck@pathai.com). PathAI will respond to these requests within 1 month of receipt.

## Code availability

Not all original code can be made publicly available. The codes for cell- and tissue-type model training, inference and feature extractions are not disclosed. To safeguard PathAI’s intellectual property, access requests for such code will not be considered. An application for a US patent for the algorithm discussed herein has been submitted (WO2022/165433). The source codes for all downstream data analyses and figure generation in this work are publicly available and can be downloaded from GitHub: https://github.com/Path-AI/AIM-NASH-DDT-manuscript.
