# Supplementary material for: AI-based automation of enrollment criteria and endpoint assessment in clinical trials in liver diseases
Source: Nat Med. 2024 Aug 7;30(10):2914–23. doi: 10.1038/s41591-024-03172-7 (PMC11485234; doi:10.1038/s41591-024-03172-7)
Supplement: Supplementary file 1 — Supplementary Tables 1–9. [file 41591_2024_3172_MOESM1_ESM.pdf]

nature medicine

Supplementary information

https://doi.org/10.1038/s41591-024-03172-7

AI-based automation of enrollment criteria and endpoint assessment in clinical trials in liver diseases

In the format provided by the authors and unedited

Supplementary Table 1. Model development and test dataset characteristics.

Dataset	Trial Phase	Number of WSIs (H&E, MT)	Drug class	Enrollment criteria	NCT registration number
Model development datasets					
1	3	2188, 2188	ASK1 inhibitor	MASH diagnosis; fibrosis F3[15]	NCT03053050
2	3	2488, 2478	ASK1 inhibitor	MASH diagnosis; fibrosis F4[15]	NCT03053063
3	2b	528, 528	Monoclonal antibody directed against LOXL2	MASH defined as steatosis > 5% with associated lobular inflammation: Ishak stage 3,4[16]	NCT01672866
4	2b	561, 554	Monoclonal antibody directed against LOXL2	MASH diagnosis; Ishak stage 5,6[17]	NCT01672879
5	2	158, 163	ASK1 Inhibitor, monoclonal antibody directed against LOXL2	Evidence of MASH with fibrosis on biopsy[18]	NCT02466516
6	2	312, 312	PPARδ agonist	Definite MASH; MAS ≥ 4 with 1 per component; fibrosis F1, F2, F3[21]	NCT03551522
7	3	1477, 766	Nucleotide analogue (antiviral)	HBV[19]	NCT00117676
8	3	851, 415	Nucleotide analogue (antiviral)	HBV[19]	NCT00116805
9	2b	331, 333	Monoclonal antibody directed against LOXL2	PSC[20]	NCT01672853
Analytic performance test set					
10	2b	639, 633	Insulin sensitizer	Definite MASH; MAS ≥ 4 with 1 per component; Fibrosis F1, F2, F3[22]	NCT02784444
11	2b	938, 945	ASK1 inhibitor, ACC inhibitor, FXR agonist	MASH diagnosis; fibrosis F3 or F4[25]	NCT03449446

ASK1, apoptosis signal-regulating kinase 1 (also known as mitogen-activated protein kinase kinase

kinase 5); ACC, Acetyl-CoA Carboxylase; F, fibrosis stage; FXR, farnesoid X receptor; HBV, hepatitis B virus; H&E, hematoxylin and eosin; LOXL2, lysyl oxidase-like 2; MT, Masson’s trichrome; MAS, metabolic dysfunction-associated steatotic liver disease Activity Score; MASH, metabolic dysfunction-associated steatohepatitis; PPARδ, peroxisome proliferator activated receptor delta; PSC, primary sclerosing cholangitis; WSI, whole slide image.

Supplementary Table 2. Algorithm repeatability assessment using 10 independent reads per WSI.

	**Number of WSIs**	**Model versus model agreement rate**
Steatosis	639	100%
Lobular inflammation	639	100%
Ballooning	639	100%
Fibrosis	633	100%

WSI, whole slide image.

Supplementary Table 3. Correlations between the AI-derived continuous scoring system and comparable noninvasive tests. Values were derived from Kendall's tau rank correlation analysis. FDR correction of p-values was performed using the Benjamini-Hochberg procedure.

**Continuous scoring system**	**NIT**	**Kendall's Tau**	***P* value**	***n***
Continuous fibrosis stage	FibroScan	0.33	2.49E–11	188
Continuous fibrosis stage	FIB4	0.23	1.56E–06	207
Continuous fibrosis stage	ELF	0.22	2.52E–06	210
Continuous fibrosis stage	TIMP1	0.11	2.01E–02	210
Continuous fibrosis stage	PIIINP	0.14	3.03E–03	210
Continuous fibrosis stage	MRI-PDFF	–0.11	2.36E–01	59
Continuous fibrosis stage	Morphometric quantitative collagen (%)	0.56	2.20E–32	205
Continuous steatosis grade	MRI-PDFF	0.52	4.83E–09	59
Continuous steatosis grade	Morphometric quantitative collagen (%)	–0.16	5.42E–04	205
Continuous lobular inflammation grade	C-reactive protein	0.13	5.04E–03	211
Continuous lobular inflammation grade	Adiponectin	–0.15	1.38E–03	211
Continuous ballooning grade	HbA1C	0.16	8.36E–04	211

AI, artificial intelligence; ELF, enhanced liver fibrosis test; FIB4, fibrosis-4; HbA1C, hemoglobin A1c; MRI-PDFF, magnetic resonance imaging derived proton density fat fraction; NIT, noninvasive test; PIIINP, procollagen III N-terminal peptide; Kendall's Tau, Kendall's rank correlation coefficient for ordinal scores; TIMP, tissue inhibitor of metalloproteinase; TIMP1, TIMP metallopeptidase inhibitor 1.

Supplementary Table 4. Deployment of AIM-MASH in retrospective analyses of Phase 2/2b clinical trial cohorts.

NCT (Phase)	Inclusion Criteria (CRN Fibrosis + MAS)	Drug Class *	N	NITs Used **	Reader Approach	Ref.	Surrogate Histologic Endpoints (SHE) + Exploratory Results		
								Met by AIM-MASH	Met by Pathologist
NCT03987451 (Phase 2)	Histologic evidence of MASH with F4 fibrosis and MAS >3	GLP-1 agonist/ insulin regulation and weight loss	BL:70 W48:70	MRI-PDFF	Single pathologist score	Poster	SHE1	Not assessed	No
							SHE2	Not assessed	No
							Exploratory	Reduction in steatosis consistent between AIM-MASH and MRI-PDFF measurement	
NCT03987451 (Phase 2)	Histologic evidence of MASH with F4 fibrosis and MAS >3	GLP-1 agonist/ insulin regulation and weight loss	BL:70 W48:70	MRI-PDFF	Single pathologist score	Poster	Exploratory	AIM-MASH reported lower placebo response than pathologist; Good agreement between AIM-MASH and pathologist scoring of change in fibrosis, inflammation, ballooning, and steatosis	
NCT02970942 (Phase 2)	Histologic evidence of MASH with fibrosis F1 to F3	GLP-1 agonist/ insulin regulation and weight loss	BL: 320 W72:251	Not assessed	Pathologist consensus score (N=2)	Poster	SHE1	Yes	Yes
							SHE2	No	No
							Exploratory	Dose dependent drug effect seen with pathologist and AIM-MASH scoring; AIM-MASH continuous scores detected significant change in fibrosis compared to placebo	
NCT03900429 (Phase 3)	Adults with ≥3 metabolic risk factors, liver stiffness	THR-β agonist/ steatosis reduction	BL: 782 W52: 777	Not assessed	Pathologist consensus score (N=2)	Reference 33<br>Poster	SHE1	Yes	Yes

	≥8.5kPa, hepatic fat ≥8%, biopsy-confirmed MASH with F1B-F3 fibrosis, and a metabolic dysfunction-associated steatohepatitis (MAS) ≥4						SHE2	Yes	Yes
							Exploratory	Not assessed	Not assessed
NCT03551522 (Phase 2)	Histologic evidence of MASH with fibrosis F1 to F3 and MAS ≥ 4 with a score of at least 1 for each MAS component	PPARδ agonist/ reduce steatosis and inflammation	BL:152	MRI-PDFF	Pathologist consensus scores (N=2)	Reference 34 Poster	SHE1	Not assessed	Not assessed
							SHE2	Not assessed	Not assessed
							Exploratory	AIM-MASH quantified MASH histologic features plus interface hepatitis and portal inflammation in BL biopsies	
NCT03486899 (Phase 2b)	Histologic evidence of MASH with fibrosis F3 and NAS score of > 1 for each MAS component	FGF21 analog/ steatosis reduction	BL:197 W24:197	MRE MRI-PDFF	Single pathologist score	Reference 35 Poster	SHE1	No	No
							SHE2	No	No
							Exploratory	AIM-MASH and pathologist scoring both showed improvement for ballooning and lobular inflammation, fibrosis in treated arm; Correlations between AIM-MASH and NITs found; AIM-MASH continuous scores showed statistically significant improvement in all MAS components from BL compared to placebo	
NCT02912260 (Phase 2)	Histologic evidence of MASH with fibrosis F1 to F3 and MAS ≥ 4 with a score of at least 1 for each MAS	THR-β agonist/ steatosis reduction	BL:104 W36:104	MRI-PDFF, FIB-4, ELF TE	Pathologist consensus score (N=2)	Reference 36 Poster	SHE1	Yes	Yes
							SHE2	Yes	Yes

	component						Exploratory	AIM-MASH continuous steatosis, ballooning, lobular inflammation scoring correlates with MRI-PDFF; AIM-MASH area of portal inflammation correlates with FIB-4, ELF, TE

SHE 1: Resolution of steatohepatitis on overall histopathological reading and no worsening of liver fibrosis on MASH CRN fibrosis score. Resolution of steatohepatitis is defined as absent fatty liver disease or isolated or simple steatosis without steatohepatitis and a MAS score of 0–1 for inflammation, 0 for ballooning, and any value for steatosis

SHE2: Improvement in liver fibrosis greater than or equal to one stage (MASH CRN fibrosis score) and no worsening of steatohepatitis (defined as no increase in MAS for ballooning, inflammation, or steatosis)

Exploratory Results: Retrospective exploratory investigation using AIM-MASH in collaboration with study sponsors
MASH, metabolic dysfunction-associated steatohepatitis; MAS, metabolic dysfunction-associated steatotic liver disease Activity Score; BL, Baseline; W, Week.

*Drug Classes
GLP-1, glucagon-like peptide-1 receptor; THR-β, thyroid hormone receptor β; FGF21, fibroblast growth factor 21; PPARδ, peroxisome proliferator activated receptor delta

**MRI-PDFF, magnetic resonance imaging proton density fat fraction; FIB-4, fibrosis-4; ELF, enhanced liver function; TE, transient elastography; MRE, magnetic resonance elastography

Supplementary Table 5. Example instructions for the interpretations of histologic features.

Histologic feature	Example instructions
Lobular inflammation	Place label regions containing at least three inflammatory cells, not including those within sinusoids. Do not label regions of portal inflammation with this region label.
Hepatocyte ballooning	Please use this label on regions of hepatocellular ballooning. Hepatocellular ballooning is defined as round cells with rarified cytoplasm that are at least 50% larger than neighboring normal cells.
Steatosis	Please use this label on regions of dense steatosis.
Thick pathologic fibrotic septa	Please use this label for thickened fibrotic septae extending from portal and central regions considered when staging liver biopsies.
Portal tract (normal)	Please use this label for normal-appearing, small-/medium-sized portal regions, not expanded by fibrosis or inflammation.
Portal tract (abnormal)	Please use this label for portal regions expanded by inflammation, fibrosis, bile ductular proliferation, or any combination of the above.
Large normal septa	Please use this label for larger intrahepatic normal septae (usually containing larger arteries, veins, and bile ducts) that would not be included when staging liver biopsies.
Subcapsular fibrosis	Please use this label for normal subcapsular regions of fibrosis not considered when staging liver biopsies.

Supplementary Table 6. AI-derived models, input substances, and objectives for application.

Model name	Major input substances	Objectives
Artifact models	Background, blur artifact, bad stain artifact, black spots, bubble, cautery, crushed tissue, hair, margin ink, marker tape, skin cell, tissue fold, rainbow pattern artifact	Remove unwanted regions of WSI that should be excluded from downstream analysis
MT tissue models	Lumen, blood vessel, bile duct, cirrhotic septal portal fibrosis, hilar fibrosis, large septal area, perisinusoidal fibrosis, septal fibrosis, subcapsular fibrosis, thick fibrotic septae, normal portal area, normal portal triad, fibrosis	Detect fibrosis regions to identify and quantify features of interest
MT large septae models	Normal portal area, normal portal triad, perisinusoidal fibrosis, thick fibrotic septae	Detect regions of pathological fibrosis
H&E tissue models	Lobular Inflammation, portal inflammation, interface hepatitis, bile duct, blood vessel, Normal hepatocytes, hepatocellular swelling, hepatocellular ballooning, steatosis, microvesicular steatosis	Detect macrovesicular steatosis, hepatocellular ballooning, and lobular inflammation regions to identify and quantify features of interest
H&E GNN models	*Overlays from H&E tissue model*: Lobular inflammation, portal inflammation, interface hepatitis, bile duct, blood vessel, normal hepatocytes, hepatocellular, swelling, hepatocellular ballooning, steatosis, microvesicular steatosis	Compute slide-level MASH CRN ordinal grades
MT GNN models	*Overlays from MT large septae model:* Large septae, pathological fibrosis, other tissue<br>*Overlays from MT tissue model:* Fibrosis, bile duct, blood vessel, other tissue	Compute slide-level MASH CRN ordinal stage

CRN, Clinical Research Network; GNN, graph neural network; H&E, hematoxylin and eosin; MT, Masson's trichrome; MASH, metabolic dysfunction-associated steatohepatitis; WSI, whole slide image

Supplementary Table 7. Description of models.

Model Name	Purpose	Model Input (during inference)	Model Output
**Segmentation Models**			
Model 1: Artifact Model	To remove unwanted regions which should not even be considered as input for other models	H&E Whole Slide Image	Output is a 3D matrix of class probabilities for each pixel in the WSI. Artifact segmentation classes: Usable tissue, Artifact area, and Background area.
Model 2: H&E Tissue Model	Detect steatosis, hepatocellular ballooning, lobular inflammation regions and other to compute features of interest	H&E Whole Slide Image	Output is a 3D matrix of class probabilities for each pixel in the WSI. H&E segmentation classes: Lobular Inflammation, Portal Inflammation, Interface Hepatitis, Bile Duct, Blood Vessel, Normal Hepatocytes, Hepatocellular Swelling, Hepatocellular Ballooning, Steatosis, Microvesicular Steatosis, Normal Interface and Other/remaining Tissue .
Model 3a: Trichrome Tissue Model	Detect fibrosis region to compute features of interest	Trichrome Whole Slide Image	Output is a 3D matrix of class probabilities for each pixel in the WSI. Trichrome segmentation classes: Collagen/Fibrosis, Bile Duct, Lumen, Blood Vessel and Other/remaining Tissue
Model 3b: Trichrome Pathological Fibrosis Model	Detect pathological fibrosis region in all fibrosis area	Trichrome Whole Slide Image	Output is a 3D matrix of class probabilities for each pixel in the WSI. Trichrome Pathological Fibrosis segmentation classes: Pathological Fibrosis, Normal Collagen and Other/remaining Tissue
**Graph Neural Networks**			
Model 4a: H&E GNN Model - Steatosis	Compute slide-level MAS ordinal scores for Steatosis	Raw model output from Model 2	Output is a single integer score for Steatosis (values in the range of 0-3)

Model 4b: H&E GNN Model - Ballooning	Compute slide-level MAS ordinal scores for Hepatocellular Ballooning	Raw model output from Model 2	Output is a single integer score for Hepatocellular Ballooning (values in the range of 0-2)
Model 4c: H&E GNN Model - Lobular Inflammation	Compute slide-level MAS ordinal scores for Lobular Inflammation	Raw model output from Model 2	Output is a single integer score for Lobular Inflammation (values in the range of 0-3)
Model 5: Trichrome GNN Model	Compute slide-level CRN ordinal score	Raw model outputs from Model 3a and Model 3b	Output is a single integer score for CRN score (values in the range of 0-4)

Supplementary Table 8. Training parameters for H&E, trichrome, and artifact models.

		**Artifact Model**	**H&E Tissue Model**	**Trichrome Tissue Model**	**Trichrome Large Septae Model**
Learning Rate Parameters	Base Learning Rate	0.001	0.0001	0.0001	0.001
	Learning Rate Scheduler	Staircase	Staircase	Staircase	Staircase
	Learning Rate Decay Factor	0.5	0.5	0.5	0.5
	Learning Rate Decay Steps	2500	10000	10000	5000
Batch Size	Train Batch Size	34	100	100	42
Batch Norm	Momentum Value	0.6	0.6	0.6	0.6
Optimizer	Optimizer Name	Adam	Adam	Adam	Adam
	Optimizer Epsilon	1e-4	1e-4	1e-4	1e-4
Dropout	Dropout Probability value	0.5	0.5	0.5	0.5

Supplementary Table 9. Training parameters for GNN models.

		GNN Steatosis	GNN Ballooning	GNN Lobular inflammation	GNN CRN Fibrosis
Learning Rate Parameters	Base Learning Rate	0.001	0.001	0.001	0.001
	Learning Rate Scheduler	Staircase	Staircase	Staircase	Staircase
	Learning Rate Decay Factor	0.8	0.8	0.8	0.8
	Learning Rate Decay Steps	200	200	200	200
Batch Size	Train Batch Size	32	32	32	32
Optimizer	Optimizer Name	Adam	Adam	Adam	Adam
	Optimizer betas	0.9 / 0.999	0.9 / 0.999	0.9 / 0.999	0.9 / 0.999
	Optimizer epsilon	1e-8	1e-8	1e-8	1e-8
Dropout	Dropout Probability value	0.5	0.5	0.5	0.5
Network architecture	Hidden features	128	128	128	128
	Layers	2	2	2	2
Mixed effect model	Bias multiplier	0.1	0.01	0.07	0.1
