# Supplementary material for: AI-based automation of enrollment criteria and endpoint assessment in clinical trials in liver diseases
Source: Nat Med. 2024 Aug 7;30(10):2914–23. doi: 10.1038/s41591-024-03172-7 (PMC11485234; doi:10.1038/s41591-024-03172-7)
Supplement: Supplementary file 2 — Reporting Summary [file 41591_2024_3172_MOESM2_ESM.pdf]

nature portfolio

Corresponding author(s): Andrew Beck

Last updated by author(s): 07-02-2024

Reporting Summary

Nature Portfolio wishes to improve the reproducibility of the work that we publish. This form provides structure for consistency and transparency in reporting. For further information on Nature Portfolio policies, see our Editorial Policies and the Editorial Policy Checklist.

Please do not complete any field with "not applicable" or n/a. Refer to the help text for what text to use if an item is not relevant to your study. For final submission: please carefully check your responses for accuracy; you will not be able to make changes later.

Statistics

For all statistical analyses, confirm that the following items are present in the figure legend, table legend, main text, or Methods section.

n/a	Confirmed	
☐	☒	The exact sample size (n) for each experimental group/condition, given as a discrete number and unit of measurement
☐	☒	A statement on whether measurements were taken from distinct samples or whether the same sample was measured repeatedly
☐	☒	The statistical test(s) used AND whether they are one- or two-sided<br>*Only common tests should be described solely by name; describe more complex techniques in the Methods section.*
☐	☒	A description of all covariates tested
☐	☒	A description of any assumptions or corrections, such as tests of normality and adjustment for multiple comparisons
☐	☒	A full description of the statistical parameters including central tendency (e.g. means) or other basic estimates (e.g. regression coefficient) AND variation (e.g. standard deviation) or associated estimates of uncertainty (e.g. confidence intervals)
☐	☒	For null hypothesis testing, the test statistic (e.g. F, t, r) with confidence intervals, effect sizes, degrees of freedom and P value noted<br>*Give P values as exact values whenever suitable.*
☒	☐	For Bayesian analysis, information on the choice of priors and Markov chain Monte Carlo settings
☒	☐	For hierarchical and complex designs, identification of the appropriate level for tests and full reporting of outcomes
☒	☐	Estimates of effect sizes (e.g. Cohen's d, Pearson's r), indicating how they were calculated

Our web collection on statistics for biologists contains articles on many of the points above.

Software and code

Policy information about availability of computer code

Data collection	AI-derived models, input substances, and objectives for application are detailed in Supplementary Table 6. The CNN image segmentation algorithms (artifact model, H&E tissue model, Trichrome tissue model, and Trichrome pathological fibrosis model) and GNN scoring algorithms (H&E GNN model - steatosis, H&E GNN model - ballooning, H&E GNN model - lobular inflammation, and Trichome GNN model) are proprietary software developed by PathAI.
Data analysis	Not all original code can be made publicly available. The code for cell- and tissue-type model training, inference, and feature extractions are not disclosed. To safeguard PathAI's intellectual property, access requests for such code will not be considered. The source code for all downstream data analyses and figure generation in this work are publicly available and can be downloaded from GitHub: https://github.com/Path-AI/AIM-NASH-DDT-manuscript.

For manuscripts utilizing custom algorithms or software that are central to the research but not yet described in published literature, software must be made available to editors and reviewers. We strongly encourage code deposition in a community repository (e.g. GitHub). See the Nature Portfolio guidelines for submitting code & software for further information.

Data

Policy information about availability of data

All manuscripts must include a data availability statement. This statement should provide the following information, where applicable:

- Accession codes, unique identifiers, or web links for publicly available datasets
- A description of any restrictions on data availability
- For clinical datasets or third party data, please ensure that the statement adheres to our policy

The histopathology data collected for this study is maintained by PathAI to preserve patient confidentiality and the proprietary image analysis. Access to histopathology features will be granted to academic investigators without relevant conflicts of interest for non-commercial use who agree not to distribute the data. Access requests can be made to Andrew Beck (andy.beck@pathai.com). Any additional information required to reanalyze the data reported in this paper relating directly to the clinical datasets (STELLAR-3, STELLAR-4, GS-US-321-0105, GS-US-321-0106, GS-US-384-1497, ENHANCE, HBV, PSC, EMMINENCE, and ATLAS datasets) will be considered at the discretion of the source institute for the clinical trial in question. Requests will be considered from academic investigators without relevant conflicts of interest for non-commercial use who agree not to distribute the data. Data requests should be sent to Andrew Beck (andy.beck@pathai.com). PathAI will respond to these requests within one month of receipt.

Research involving human participants, their data, or biological material

Policy information about studies with human participants or human data. See also policy information about sex, gender (identity/presentation), and sexual orientation and race, ethnicity and racism.

Reporting on sex and gender	The results presented in our manuscript are retrospective analyses of completed clinical trials of patients with MASH. While the research, therefore, did involve specimens from human subject participants, no sex- or gender-based analyses were performed herein as these were outside the scope of the current study.
Reporting on race, ethnicity, or other socially relevant groupings	The results presented in our manuscript are retrospective analyses of completed clinical trials of patients with MASH. While the research, therefore, did involve specimens from human subject participants, no analyses of race, ethnicity, or other socially relevant groupings were performed herein as these were outside the scope of the current study.
Population characteristics	Anonymized liver tissue samples and digitized WSI of hematoxylin and eosin (H&E)- and trichrome-stained liver biopsies were obtained from adult patients in the following clinical datasets:<br>STELLAR-3 (Reference 11): Patients were diagnosed with MASH (F3 fibrosis) and were treated with selonsertib (an ASK1 inhibitor)<br>STELLAR-4 (Reference 11): Patients were diagnosed with MASH (F4 fibrosis) and were treated with selonsertib (an ASK1 inhibitor)<br>GS-US-321-0105 (Reference 12): Patients were diagnosed MASH with bridging fibrosis and were treated with simtuzumab (a monoclonal antibody directed against LOXL2)<br>GS-US-321-0106 (Reference 13): Patients were diagnosed with MASH with compensated cirrhosis and were treated with simtuzumab (a monoclonal antibody directed against LOXL2)<br>GS-US-384-1497 (Reference 14): Patients were diagnosed with MASH and were treated with selonsertib (an ASK1 inhibitor) with or without simtuzumab (a monoclonal antibody directed against LOXL2)<br>ENHANCE (Reference 17): Patients were diagnosed with MASH (MAS >= 4 and F1-F3 fibrosis) and were treated with seladelpar (a PPAR delta agonist)<br>HBV (Reference 15): Patients were diagnosed with hepatitis B (HBV) and were treated with tenofovir disoproxil fumarate (a nucleotide analogue)<br>PSC (Reference 16): Patients were diagnosed with PSC and were treated with simtuzumab (a monoclonal antibody directed against LOXL2)<br>EMMINENCE (Reference 21): Patients were diagnosed with MASH (MAS >= 4 and F1-F3 fibrosis) and were treated with MSDC-0602K (an insulin sensitizer)<br>ATLAS (Reference 22): Patients were diagnosed with MASH (F3 or F4 fibrosis) and were treated with a combination of selonsertib (an ASK1 inhibitor), firsocostat (an ACC inhibitor), and cilofexor (an FXR agonist)
Recruitment	Anonymized liver tissue samples and digitized WSI of hematoxylin and eosin (H&E)- and trichrome-stained liver biopsies were obtained from adult patients with MASH that had in participated in any of the following complete randomized controlled trials of MASH therapeutics: NCT03053050 [11], NCT03053063 [11], NCT01672866 [12], NCT01672879 [13], NCT02466516 [14], NCT03551522 [17], NCT00117676 [15], NCT00116805 [15], NCT01672853 [16], NCT02784444 [22], NCT03449446 [23]. Recruitment information for each trial is described the study referenced for each clinical trial.
Ethics oversight	Anonymized liver tissue samples and digitized WSI of hematoxylin and eosin (H&E)- and trichrome-stained liver biopsies were obtained from adult patients with MASH that had in participated in any of the following complete randomized controlled trials of MASH therapeutics: NCT03053050 [11], NCT03053063 [11], NCT01672866 [12], NCT01672879 [13], NCT02466516 [14], NCT03551522 [17], NCT00117676 [15], NCT00116805 [15], NCT01672853 [16], NCT02784444 [22], NCT03449446 [23]. Ethics oversight, including approval by central institutional review boards and description of informed consent for future research and tissue histology, is described the study referenced for each clinical trial.

Note that full information on the approval of the study protocol must also be provided in the manuscript.

Field-specific reporting

Please select the one below that is the best fit for your research. If you are not sure, read the appropriate sections before making your selection.

Life sciences   Behavioural & social sciences   Ecological, evolutionary & environmental sciences

For a reference copy of the document with all sections, see nature.com/documents/nr-reporting-summary-flat.pdf

Life sciences study design

All studies mu

Sample si

Machine learning (ML) model development and external, held-out test sets are summarized in Supplementary Table 1. ML models for segmenting and grading/staging nonalcoholic steatohepatitis (NASH) histologic features were trained using 8747 haematoxylin and eosin (H&E) and 7660 Masson's trichrome (MT) WSIs from six completed phase 2b and phase 3 MASH clinical trials, covering a range of drug classes, trial enrollment criteria, and patient statuses (screen fail versus enrolled) (Supplementary Table 1). H&E and MT liver biopsy WSIs from primary sclerosing cholangitis (PSC) and chronic hepatitis B infection were also included in model training. The latter dataset enabled the models to learn to distinguish between histologic features that may visually appear to be similar but are not as frequently present in MASH (e.g. interface hepatitis), in addition to enabling coverage of a wider range of disease severity than is typically enrolled in MASH clinical trials.

While no a prori sample size calculation was performed herein, all data used to evaluate the utility of our model were obtained retrospectively from clinical trials that were determined to be appropriately powered to detect differences in treatment response prior to trial initiation.

Data exclu

Replicatior

Model performance repeatability assessments and accuracy verification were conducted in an external, held-out validation dataset (Analytic performance test set) comprising WSIs of baseline and end of treatment (EOT) biopsies from a completed phase 2b MASH clinical trial (Supplementary Table 1). Repeatability of AI-based scoring (intra-method variability) was assessed by deploying the present AI algorithms on [illegible]

Randomiz

The model development dataset described above was split into training (~70%), validation (~15%), and held-out test (~15%) sets. The dataset was split at the patient level, with all WSIs from the same patient allocated to the same development set. Sets were also balanced for key MASH disease severity metrics, such as MASH CRN steatosis grade, ballooning grade, lobular inflammation grade, and fibrosis stage, to the greatest extent possible. The balancing step was occasionally challenging because of the MASH clinical trial enrollment criteria, which restricted the patient population to those fitting within specific ranges of the disease severity spectrum. The held-out test set contains a

Blinding

Pathologists and the algorithm scored each WSI independently, without knowledge of other scores given.

Reporting for specific materials, systems and methods

We require information from authors about some types of materials, experimental systems and methods used in many studies. Here, indicate whether each material, system or method listed is relevant to your study. If you are not sure if a list item applies to your research, read the appropriate section before selecting a response.

Materials & experimental systems

n/a Involved in the study

- Antibodies
- Eukaryotic cell lines
- Palaeontology and archaeology
- Animals and other organisms
- Clinical data
- Dual use research of concern
- Plants

Methods

n/a Involved in the study

- ChIP-seq
- Flow cytometry
- MRI-based neuroimaging

Clinical data

Policy information about clinical studies

All manuscripts should comply with the ICMJE guidelines for publication of clinical research and a completed CONSORT checklist must be included with all submissions.

Clinical trial registration   Clir

Study protocol   *Note where the full trial protocol can be accessed OR if not available, explain why.*

Data collection | *Describe the settings and locales of data collection, noting the time periods of recruitment and data collection.*

Outcomes | *Describe how you pre-defined primary and secondary outcome measures and how you assessed these measures.*

his checklist template is licensed under a Creative Commons Attribution 4.0 International License, which permits use, sharing, adaptation, distribution and reproduction in any medium or format, as long as you give ppropriate credit to the original author(s) and the source, provide a link to the Creative Commons license, and indicate if changes were made. The images or other third party material in this article are included in e article's Creative Commons license, unless indicated otherwise in a credit line to the material. If material is not included in the article's Creative Commons license and your intended use is not permitted by atutory regulation or exceeds the permitted use, you will need to obtain permission directly from the copyright holder. To view a copy of this license, visit http://creativecommons.org/licenses/by/4.0/
